# Clinicopathological features of desmoplastic small round cell tumors: clinical series and literature review

**DOI:** 10.1186/s12957-021-02310-6

**Published:** 2021-06-30

**Authors:** Ling-Ling Wang, Zhong-He Ji, Ying Gao, Hong Chang, Ping-Ping Sun, Yan Li

**Affiliations:** 1 Department of Pathology, Beijing Shijitan Hospital, Capital Medical University, Beijing, 100038 People’s Republic of China; 2 Department of Peritoneal Cancer Surgery, Beijing Shijitan Hospital, Capital Medical University, No 10, Tieyi Road, Yangfangdian Street, Haidian District, Beijing, 100038 People’s Republic of China

**Keywords:** Desmoplastic small round cell tumor, Pathology, EWSR1-WT1, Desmin, Vimentin

## Abstract

**Background and purpose:**

Desmoplastic small round cell tumor (DSRCT) is a highly malignant sarcoma that occurs in the abdominopelvic cavities of adolescents. The accurate diagnosis of DSRCT is challenging owing to limited literatures. Our study aimed to investigate the relationship between clinicopathological features and prognosis in patients with DSRCTs.

**Methods:**

Data of 8 patients with DSRCT originating from the abdominal cavity were retrospectively reviewed. The clinical manifestations, pathological characteristics, treatment approaches, and prognosis were analyzed. The histopathological (identified using hematoxylin-eosin staining), immunohistochemical, and molecular diagnostic (using fluorescence in situ hybridization) features were also reviewed.

**Results:**

All patients were male aged between 24 and 45 years (median age, 30 years). The main clinical symptoms included abdominal distension, abdominal pain, and constipation. Seven of the 8 patients developed metastases to either distant organs or lymph nodes. Multiple gray nodules with diameters of 1–10 cm and poorly defined boundaries were scattered throughout the omentum and mesentery. Histopathological examination demonstrated well-defined nests composed of small round blue cells separated by markedly desmoplastic stroma. Immunohistochemical analysis revealed positive expressions of desmin, vimentin and C-terminal of Wilm’s tumor suppressor (WT-1). The Ewing sarcoma breakpoint region 1 gene fused with WT1 (*EWSR1-WT1*) gene fusion was detected in all patients. Cytoreductive surgery (CRS) was performed in 6 patients. Follow-up period ranged from 7.5 to 28.5 months with a median of 17.2 months. Three patients died during follow-up.

**Conclusion:**

DSRCT is highly aggressive and presents distinctive morphological features. CRS is the essential therapy for DSRCT. A test for the combined expression of desmin, cytokeratins, and C-terminal of WT-1, as well as the analysis of morphologic features, might be helpful during DSRCT diagnosis, and the *EWSR1-WT1* gene fusion is the gold standard for definitive diagnosis. Our work will provide new insights into the diagnosis and treatment of DSRCTs.

## Background

Desmoplastic small round cell tumor (DSRCT) is a highly malignant sarcoma, which commonly occurs in the abdominopelvic cavities of adolescents. DSRCT usually occurs in young men aged 20–30 years, accounting for 85–90% of total cases [1]. This tumor was first described by Gerald and Rosei in 1989 [2] and got its official name in 1991 [3]. Owing to limited literature published on DSRCT, making an accurate diagnosis has been challenging. Most patients, therefore, present to hospitals with advanced stage of the disease.

Although the tissue of origin and clinical symptoms have not yet been specified for DSRCT, its pathological morphological features, immunohistochemical (IHC) biomarkers, and molecular properties are relatively distinct. Generally, tumor cells exhibit multi-lineage differentiation and diverse morphology and may express epithelial, mesenchymal, and neural markers [4]. Representative features of DSRCT are large tumors in the abdominal cavity accompanied by numerous smaller peritoneal implants. This tumor can also occur in rare locations such as the para-testicular region, pleura, posterior cranial fossa, bone, soft tissue, ovary, parotid gland, or lung [5–12]. Since most patients remain asymptomatic until the tumor burden is high, many are clinically diagnosed when their disease is in the advanced stage, and some patients may only be incidentally diagnosed during imaging examinations for other diseases.

In the present study, we aimed to elucidate clinical and pathological characteristics of DSRCT and perform relevant literature review.

## Methods

### Clinical information

This was a retrospective study on 8 DSRCT patients (7 inpatients and 1 outpatient) diagnosed between January 2012 and November 2019 at the Department of Pathology, Beijing Shijitan Hospital, Capital Medical University. Clinicopathologic information was obtained from archived medical records. This study was approved by the Ethics Committee of Institutional Review Board and was conducted according to the Declaration of Helsinki. Informed consent was obtained from all patients.

### Surgery-based integrated treatment

All patients underwent comprehensive surgical-based treatments, including laparotomy, cytoreductive surgery (CRS) plus hyperthermic intraperitoneal chemotherapy (HIPEC), and perioperative chemotherapy. Combined CRS and HIPEC was performed by a designated team specialized in peritoneal cancer therapy. Laparotomy was performed under general anesthesia, with a midline incision from the xiphoid to the pubis. Tumor size and location were then determined. The extent of tumor spread and invasiveness in the abdominopelvic cavity were thoroughly explored and evaluated using the peritoneal cancer index (PCI) [12]. Subsequently, maximal CRS was performed, including the curative or palliative resection of primary tumors with acceptable margins, resection of any involved adjacent structures, lymphadenectomy, and peritonectomy [13]. Completeness of cytoreduction (CC) was evaluated based on the estimated size of residual tumors [14].

HIPEC was performed using the open coliseum technique. This technique involved dissolving each drug in heated saline at 43°C ± 0.5°C and infusing the solution into the cavity for 30 min with a flow rate of 400 ml/min. Standard HIPEC regimens consist of either cisplatin (120 mg) plus mitomycin (30 mg) or cisplatin (120 mg) plus docetaxel (120 mg). After HIPEC, the digestive tract, urinary tract, and/or intestinal stoma were reconstructed where necessary. Abdominal drainage tubes were placed, and the incision was sutured with reduced tension.

### Definition of PCI or CC

**Fig. 1:**
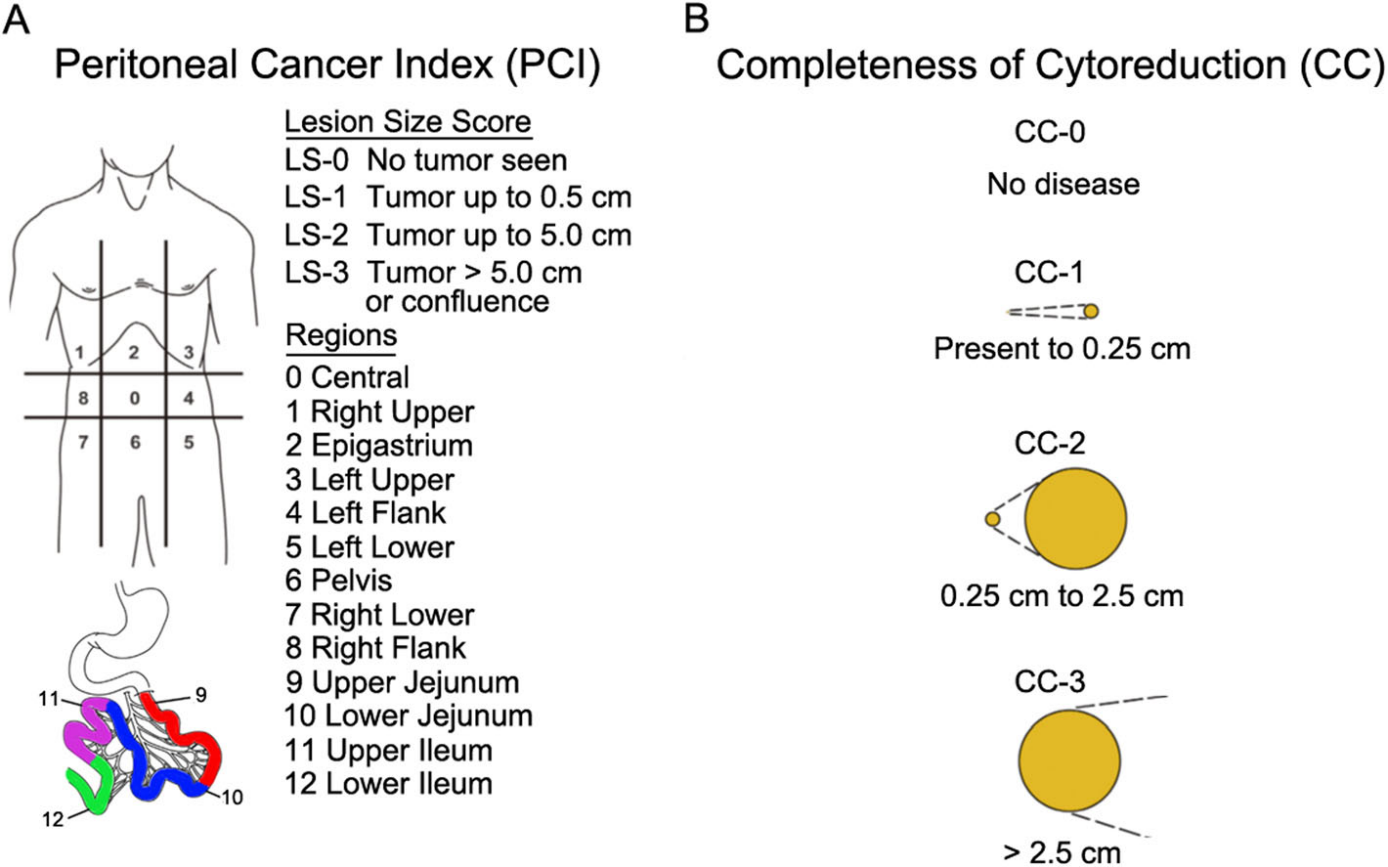
Illustration of peritoneal cancer index (PCI) (**A**, left) and completeness of cytoreduction score (CC) (**B**, right)

The abdominopelvic cavity was divided into 9 regions, while the small bowel was divided into 4 regions (upper ileum, lower ileum, upper jejunum, and lower jejunum). In each of the 13 regions, PCI [15] was scored as follows (Fig. 1A): tumor volume was scored as LS0 when no visible tumor was detected; LS1 when tumor nodules were <0.5 cm in diameter; LS2 when tumor nodules were 0.5–5.0 cm in diameter; and LS3 when tumor nodules were >5 cm in diameter or when confluent lesions were detected. Summing up the scores achieved a maximum of 39 points.

CC was scored as follows (Fig. 1B): CC0 when no residual tumor was detected after CRS; CC1 when residual tumor was <0.25 cm in diameter; CC2 when residual tumor was 0.25-–2.5 cm in diameter; and CC3 when residual tumor was >2.5 cm in diameter. CC0-1 was considered as complete CRS and CC2-3 as incomplete CRS.

### Histopathology

All available hematoxylin-eosin-stained slides were independently reviewed by 2 senior pathologists. For IHC analysis, the 2-step Envision technique was used. Primary antibodies included cytokeratin (CK), epithelial membrane antigen (EMA), vimentin, synapsin (Syn), CgA, CD56, S-100, CD99, CD117, CD34, Dog-1, desmin, WT-1 N-terminal, WT-1 C-terminal, Ki-67, and p53 (Table 1). Phosphate buffer saline was used as the negative control, while the corresponding tissue was set as the positive control. Cytoplasmic markers (CK, EMA, vimentin, Syn, CgA, CD56, S-100, CD99, CD117, CD34, Dog-1, WT-1 N-terminal and desmin) and nuclear markers (Ki-67, p53, and WT-1 C-terminal) were included.

**Table 1:** Immunohistochemical panel

Antibody	Supplier	Clone	Dilution
CK	Zhongshan Golden Bridge Biology Co., Ltd. Beijing, China	AE1/AE3	1:100
EMA	Zhongshan Golden Bridge Biology Co., Ltd. Beijing, China	UMAB57	1:200
Vimentin	Zhongshan Golden Bridge Biology Co., Ltd. Beijing, China	UMAB159	1:120
Syn	Zhongshan Golden Bridge Biology Co., Ltd. Beijing, China	UMAB112	Ready to use
CgA	Zhongshan Golden Bridge Biology Co., Ltd. Beijing, China	LK2H10	Ready to use
CD56	Zhongshan Golden Bridge Biology Co., Ltd. Beijing, China	UMAB83	Ready to use
S-100	Zhongshan Golden Bridge Biology Co., Ltd. Beijing, China	poly	Ready to use
CD99	Gene Tech Co., Ltd., Shanghai, China	013	Ready to use
CD117	Roche Biology Co., Ltd.	9.7	Ready to use
CD34	Zhongshan Golden Bridge Biology Co., Ltd. Beijing, China	10C9	Ready to use
DOG1	Zhongshan Golden Bridge Biology Co., Ltd. Beijing, China	OTI1C6	Ready to use
Desmin	Zhongshan Golden Bridge Biology Co., Ltd. Beijing, China	EP15	Ready to use
WT-1 N-terminal	Gene Tech Co., Ltd., Shanghai, China	6F-H2	Ready to use
WT-1 C-terminal	Maixin Biology, Co., Ltd. Fujian, China	MX012	Ready to use
Ki-67	Zhongshan Golden Bridge Biology Co., Ltd. Beijing, China	UMAB107	1:100
P53	Zhongshan Golden Bridge Biology Co., Ltd. Beijing, China	DO-7	Ready to use

Fluorescence in situ hybridization (FISH) was used to detect the Ewing sarcoma breakpoint region 1 gene fused with Wilm’s tumor suppressor (*EWSR1-WT1*) fusion gene in paraffin-embedded tissue samples. The *EWSR1-WT1* fusion probe was purchased from AnBiPing Biotechnology (Guangzhou, China). *WT1* and *EWSR1* gene loci were represented by red (R) and green (G) signals, respectively, and the fusion was represented by red-green-merged signals (F). 1R1G1F was considered a typical positive signal, while 2R1G1F, 1R2G1F, and more than 1F were also considered positive signals. A cell was considered positive when a higher proportion of typical positive signals were present. In contrast, 2R2G was considered negative. A definite diagnosis was made when >10% in a 200-cell population was positive.

## Results

### Clinical characteristics

All patients were male, and the median age of the patients was 30 years (range 24–45 years). Initial clinical symptoms included abdominal distension (8/8, 100.0%), abdominal pain (7/8, 87.5%), altered bowel habits (2/8, 25.0%), and constipation (2/8, 25.0%). None of the patients had a family history of cancer or a history of surgery. Five patients were administered chemotherapy before surgery (62.5%). Three cases refused preoperative chemotherapy owning to poor financial status. Five patients had ascites (62.5%) with average volume of 900 ml (range 100–2200 ml). The median PCI score was 30.5 (range 7–39). The CC scores were CC0 in one patient (12.5%), CC1 in 2 (25.0%), and CC2-3 in 5 (62.5%). Six patients (75%) underwent HIPEC. Five patients (62.5%) were administered postoperative adjuvant chemotherapy. Two cases transferred to other hospitals after surgery and refused to provide chemotherapy information during follow-up. The other 1 case refused chemotherapy because he failed to benefit from preoperative chemotherapy. Clinical features of the patients are listed in Table 2.

**Table 2:** Clinical data of 8 cases of DSRCT

No	Gender/age	Symptoms	Preoperative chemotherapy	Surgical approach	Ascites (ml)	PCI/CC (Score)	Postoperative chemotherapy	Follow-up (Month)	Survival Status
1	Male/33	Altered bowel habits, abdominal distension and pain	AI solution × 2 cycles	CRS+HIPEC CRS: hepatic round ligament, greater omentum, lesser omentum, abdominal wall tumors, sub mesenteric vein root tumors, transverse colon tumors, left popliteal tumors, ascending colon surface tumors, transverse colon bowel lip, rectum + sigmoid colon, partial ileum + colon HIPEC: doxorubicin 40 mg + ifosfamide 3 g, 43 °C, 60 min	200	30/3	None	8.4	Live
2	Male/41	Stomachache, abdominal distension and pain	None	CRS+HIPEC; CRS: right colon, omentum; HIPEC: cisplatin 120 mg + docetaxel 120 mg, 43 °C, 90 min	None	7/0	ICE × 8 cycles	20.0	Live
3	Male/24	Abdominal distension, right lower quadrant pain, altered bowel habits	VAC × 4 cycles, IE × 3 cycles, irinotecan + recombinant human endostatin + anlotinib × 10 cycles	CRS+HIPEC CRS: greater omentum, right diaphragm, hepatic hilum tumor, right lobe tumor, rectum + pelvic tumor, mesenteric tumor HIPEC: doxorubicin 120 mg + ifosfamide 3 g, 43 °C, 60 min	1000	24/1	AIM plan + PD1 (details unknown)	20.3	Live
4	Male/29	Abdominal pain and distension, constipation	TC plan × 1 cycle AI solution × 2 cycles	CRS+HIPEC CRS: tumors of the upper abdominal wall, tumors of the left lower abdomen, colonic spleen flexure peritoneum, omental tumors, descending colon peritoneal tumors, hepatic ligament HIPEC: docetaxel 120 mg + ifosfamide 3 g, 43 °C, 60 min	2200	39/3	AI × 6 cycles IE × 6 cycles	14.4	Dead
5	Male/27	Abdominal pain and distension, constipation	AI Plan×7 cycles	CRS+HIPEC CRS: hepatic round ligament, greater omentum, rectal pelvic floor tumor, liver and kidney crypt tumor, small intestinal mesenteric tumor, colon mesenteric tumor, right diaphragm muscle tumor, ileocecal + appendix, left diaphragm muscle tumor HIPEC: docetaxel 120 mg + ifosfamide 3 g, 43 °C, 60 min	100	25/1	AI solution × 6 cycles + Retroperitoneal lymph node radiotherapy	28.5	Dead
6	Male/45	Abdominal pain and distension, exhaustion, fever	None	Laparotomy: partial abdominal tumor resection	Unknown	38/2	Unknown	22.6	Live
7	Male/26	Abdominal distension with nausea and vomiting	None	CRS+HIPEC CRS: omental tumor, liver curvature tumor, splenic curvature tumor HIPEC: docetaxel 120 mg, 43 °C, 30 min	1900	39/2	Unknown	8.9	Dead
8	Male/ 31	Abdominal pain and distension	IE×1cycles	Laparotomy: partial abdominal tumor resection	None	31/2	VAC solution × 5 cycles	7.5	Live

*CRS*, cytoreductive surgery; *HIPEC*, hyperthermic intraperitoneal chemotherapy; *AI*, doxorubicin + ifosfamide; *ICE*, cis-platinum + ifosfamide + etoposide; *VAC*, vincristine + pharmorubicin + cyclophosphamide; *IE*: cis-platinum + etoposide; *TC*, Taxol + carboplatin; *DOX*, doxorubicin; *DOC*, docetaxel; *IFO*, ifosfamide; *CDDP*, cis-platin; *PD-1*, Programmed cell death-1

### Medical imaging features

**Fig. 2:**
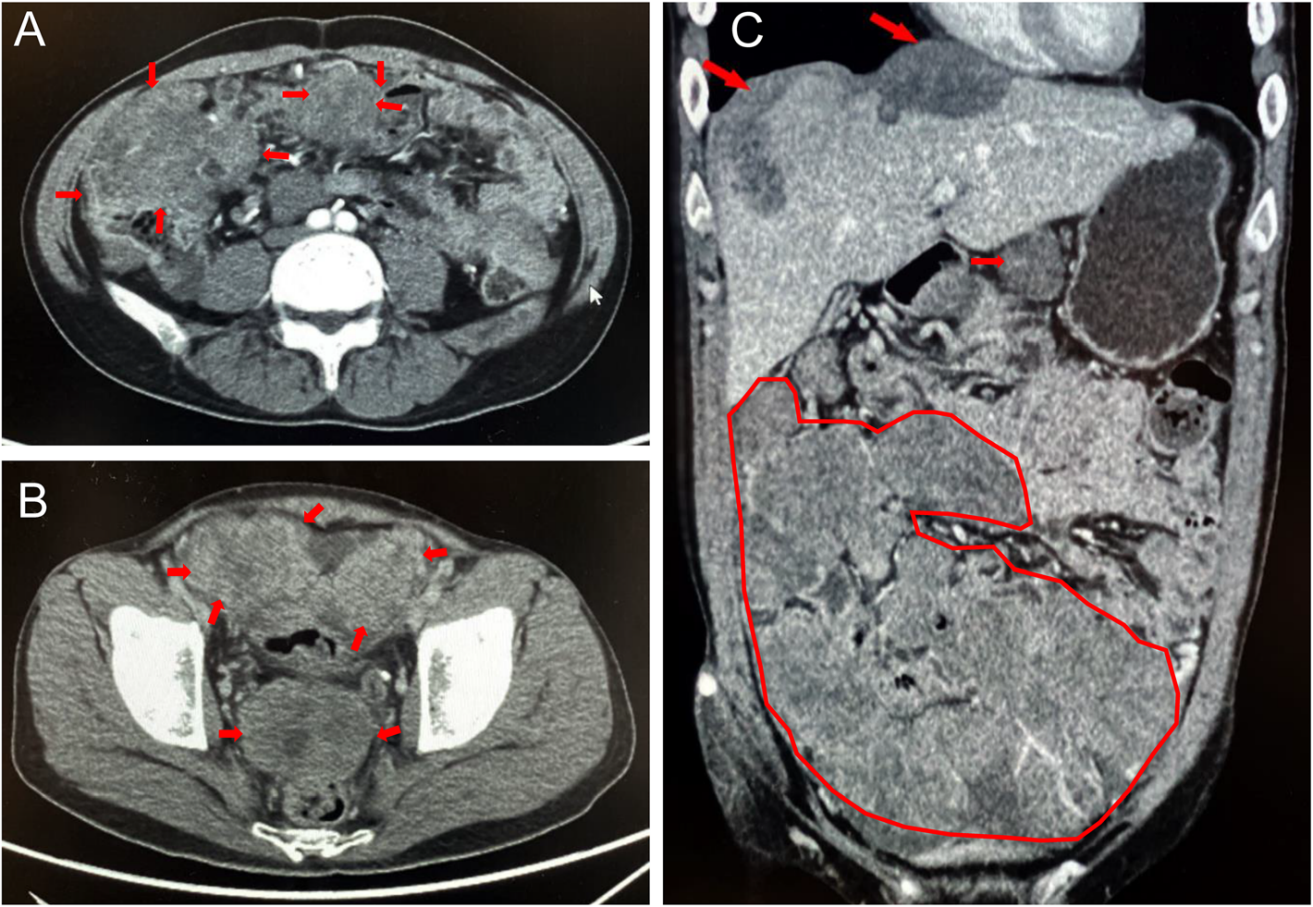
Manifestations of desmoplastic small round cell tumor in enhanced computed tomography: **A** tumor mass of mesentery and greater omentum (red arrows), **B** tumor mass in pelvic cavity (red arrows), and **C** tumor mass of mesentery (red box), greater omentum, (red box), less omentum (red arrows), and diaphragm (red arrows) in sagittal view

All patients underwent computed tomography of the abdominopelvic cavity. Multiple nodular soft tissue masses with poorly defined boundaries were located on the omentum and mesentery. The signals were unevenly enhanced (Fig. 2A–C). Multiple nodules were also observed in the liver (5/8), lungs (1/8), hilum of the spleen (1/8), hydronephrosis (1/8), and adrenal glands (1/8).

### General pathological characteristics

**Fig. 3:**
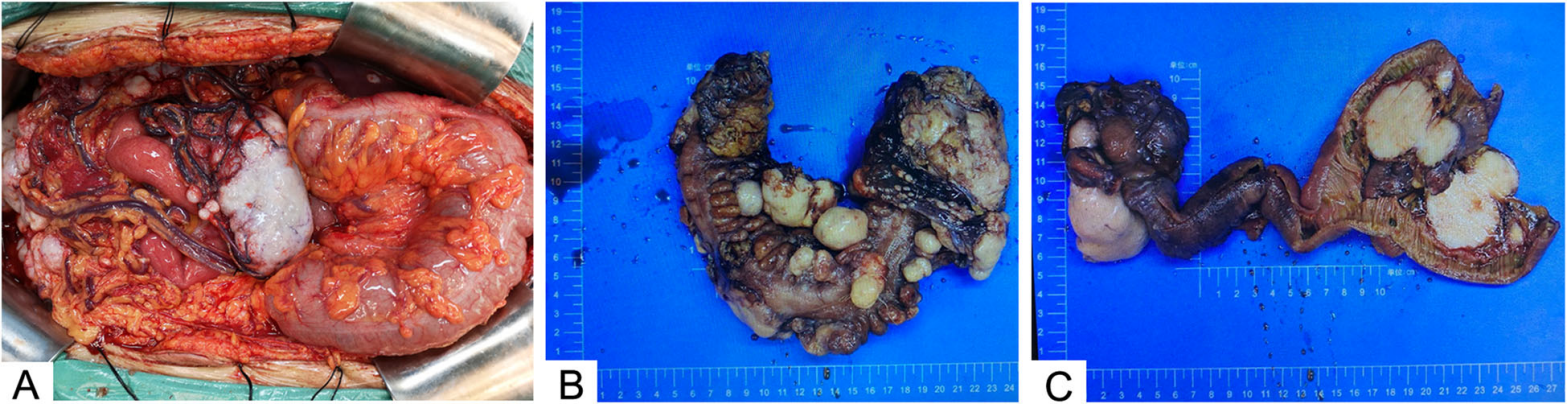
Morphology of representative desmoplastic small round cell tumor. **A** Milky white masses identified during operation in case 1. Tumor nodules of various sizes and morphology were scattered throughout the abdominopelvic cavity. **B** Multi-nodular or lobulated, gray-white masses located on the mesentery in case 1. **C** Solid tumor nodules with varying size, gray cutting surface, and medium in quality in case 2

Seven patients presented with multiple pelvic and abdominal nodules. The lesions manifested as gray-white, multi-nodular, and lobulated masses on the omentum and mesentery, spreading along the serosa to the pelvis and peritoneum (Fig. 3A, B). The sizes of implanted nodules varied with the largest diameters ranging between 1 and 10 cm. Tumors affected the small and large intestines by invading the submucosa (Fig. 3C). In one case, a single large tumor in the colonic serosa was accompanied by multiple omental nodules. The dissected surface was mostly gray and solid. Focal hemorrhage, necrosis, and cyst formation were occasionally observed.

### Histopathology features

**Fig. 4:**
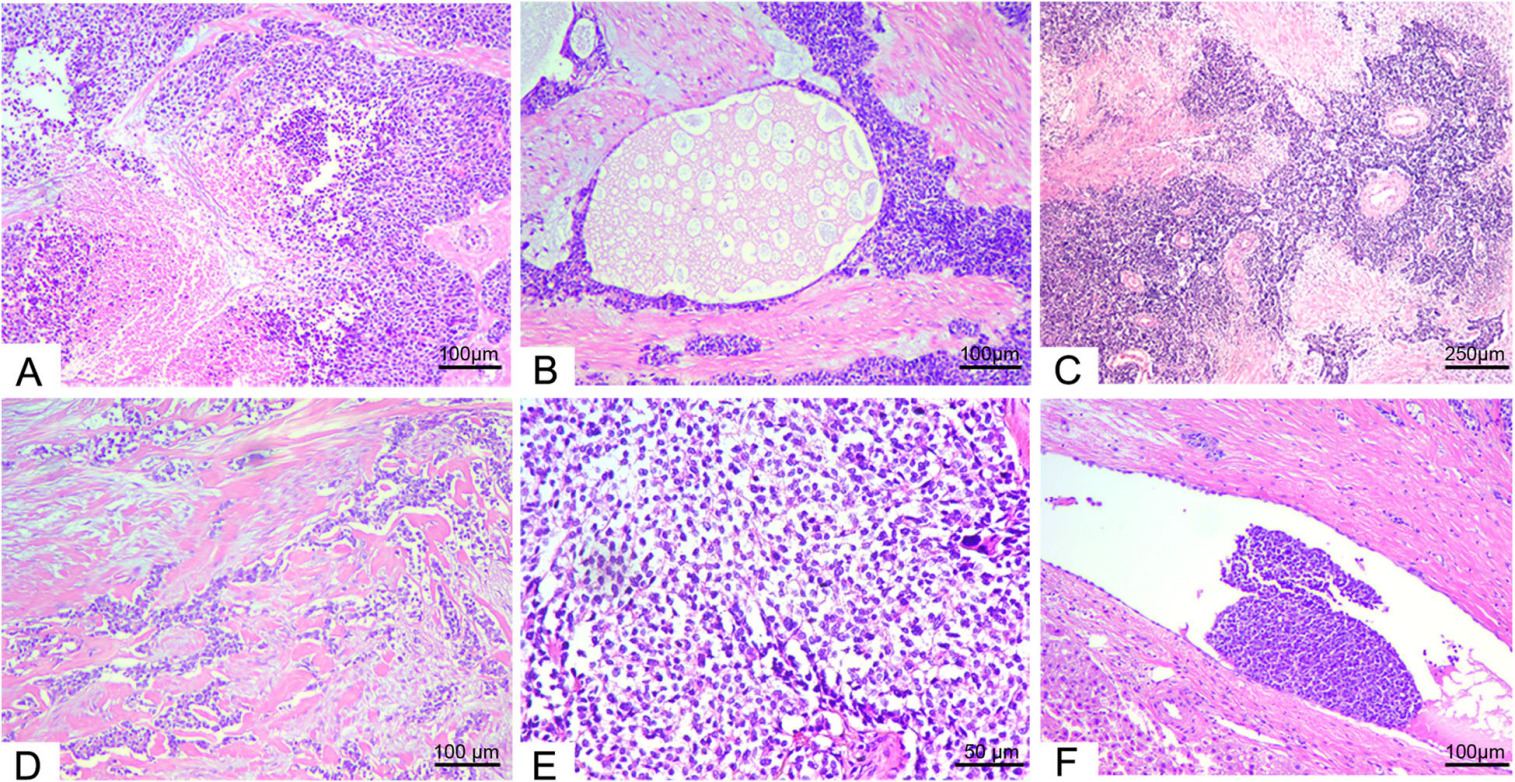
Hematoxylin-eosin staining was performed in 8 cases and representative presentations of various tumors were shown. **A** Focal necrosis in case 4 (×100). **B** Obvious cystic formation with different sizes in the cystic cavities of case 4 (×100). C Tumor cells arranged in pseudorosette-like clusters around thick-walled blood vessels in case 3 (× 40). **D** Dense interstitial fibrous connective tissue in case 3 (× 100). **E** Tumor cells with ambiguous boundaries, scarce cytoplasm, round or oval deep-staining nuclei, and obscured nucleoli in case 3 (× 200). **F** Vascular tumor embolus in case 3 (× 100)

Most tumors were composed of small round cell nests of different sizes and irregular shapes. Focal necrosis or cystic formation presented centrally in large tumor cell nests (Fig. 4A, B). Arrangements of tumor cells varied from single rows to cords, beam-like, or follicles and pseudorosette-like clusters (Fig. 4C). Most of the mesenchyme was composed of dense hyalinized fibrous tissue (Fig. 4D). Loose fibrous tissue accompanied by mucoid degeneration was also observed. Tightly arranged tumor cells with unclear borders were present. The cytoplasm was scarce and translucent or had a signet ring-like appearance, while the nuclei were round to oval and darkly stained with obscured nucleoli (Fig. 4E). Blood and lymphatic vessel invasions were observed (Fig. 4F). Invasion via the intestinal wall to the submucosa was also demonstrated. The morphological characteristics of all cases are summarized in Table 3.

**Table 3:** The morphological characteristics of all cases

Case no.	Necrosis	Cystic structure	Solid: mesenchyme	Signet ring cells	Motosis	Vascular tumors bolt	Lymph node metastasis
1	No	No	>1	Yes	<5/10HPF	Yes	Yes
2	Yes	No	<1	No	<5/10HPF	No	No
3	No	Yes	>1	Yes	>5/10HPF	Yes	Yes
4	Yes	Yes	>1	Yes	>5/10HPF	No	No
5	No	Yes	>1	No	<5/10HPF	Yes	Yes
6	No	No	<1	No	>5/10HPF	No	Yes
7	Yes	No	>1	Yes	>5/10HPF	Yes	Yes
8	No	No	>1	No	<5/10HPF	Yes	No

### IHC and molecular pathology features

**Fig. 5:**
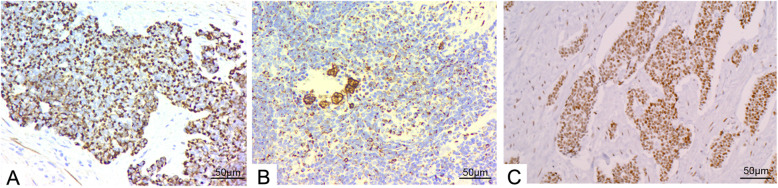
Immunohistochemistry staining was performed in 8 cases and representative presentations in case 5 were shown. **A** Characteristic focally positive expressions of desmin next to the nucleus (× 200). **B** Characteristic focally positive expressions of vimentin next to the nucleus (× 200). **C** Diffuse positive expression of C-terminal of WT1 (× 200)

Tumor cells showed characteristic paranuclear dot-like positive signals for desmin and vimentin. In 7 patients, EMA, desmin (Fig. 5A), and vimentin (Fig. 5B) were present either diffusely or focally. WT1 C-terminal was diffusely positive in 8 patients (Fig. 5C). WT1 N-terminal was focally positive in 2 patients. Syn was focally positive in 2 patients. CD56 was focally or diffusely positive in 3 patients. CgA was diffusely positive in 2 patients. CK was diffusely or focally positive in 6 patients. CD99 was diffusely positive in 5 patients. Ki-67 index ranged from 20 to 80%. S100, CD117, CD34, and Dog-1 were negative (Table 4).

**Table 4:** Immunohistochemical features of 8 cases of DSRCT

Case No.	CK	EMA	Vimentin	Syn	CgA	CD56	S-100	CD99	CD117	CD34	Dog-1	Desmin	WT-1 (C-terminal)	WT-1(N-terminal)	Ki-67	p53
1	+	-	-	Focal+	-	+	-	+	-	-	-	+	80%+	-	40%+	Mutant
2	Focal+	Focal+	+	-	-	-	-	+	-	-	-	Focal+	90%+	Focal+	20%+	Wild type
3	+	Focal+	Focal+	-	-	Focal+	-	+	-	-	-	Focal+	70%+	-	80%+	Wild type
4	+	+	Focal+	Focal+	-	Focal+	-	+	-	-	-	+	80%+	-	50%+	Mutant
5	-	+	+	-	+	-	-	+	-	-	-	+	70%+	-	60%+	Wild type
6	-	+	+	NA	+	NA	NA	NA	NA	NA	-	-	80%+	Focal+	60%+	NA
7	+	+	+	-	-	NA	NA	NA	NA	NA	-	+	70%+	NA	80%+	NA
8	+	+	+	-	-	-	-	-	-	-	-	+	90%+	-	50%+	NA

*NA*, not applicable

**Fig. 6:**
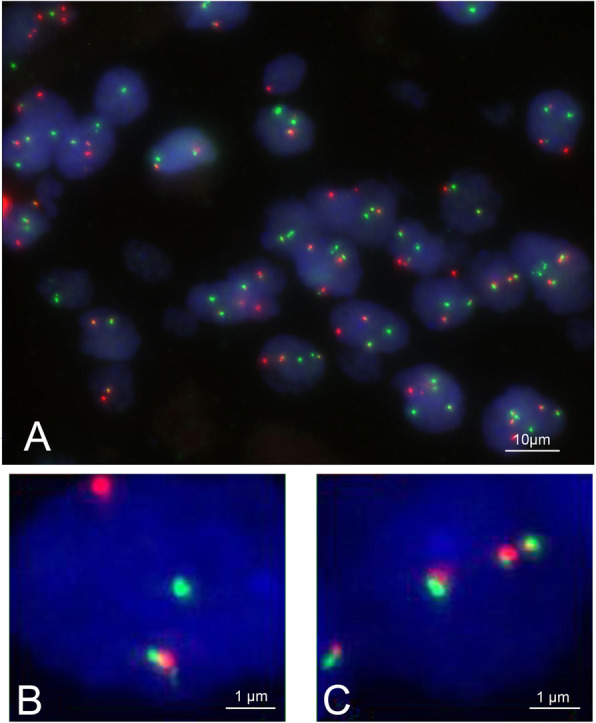
Fluorescency in situ hybridization with an EWSR1-WT1 fusion probe detected more than 10% of EWSR1-WT1 gene fusion. WT1 and EWSR1 gene loci were represented by red (R) and green (G) signals, respectively, and fusion was represented by red-green fused signals (yellow, F). n = 8. **A** Rearrangement of the EWSR1-WT1. **B**, **C** Fusion of red and green signal patterns in different cells

FISH results indicated that the *EWSR1-WT1* gene fusion was present in all patients (Fig. 6). The percent of *EWSR1-WT1* gene fusion was among 32 to 50% in all patients (Table 5).

**Table 5:** The EWSR1-WT1 fusion features in 8 cases of DSRCT detected by fluorescence in situ hybridization

Case No.	1R1G1F	Other fusion	EWSR1-WT1 fusion percent (%)
1	30	65	47.5
2	68	14	41
3	58	20	39
4	40	24	32
5	70	30	50
6	48	26	37
7	45	31	38
8	80	14	47

### Follow-up and clinical outcome

All patients were regularly followed up by either outpatient consultation or telephone interview. At a median follow-up of 17.2 months (range 7.5–28.5 months), 3 patients died. The median overall survival time was 22.9 months (95% confidence interval 14.9–30.8).

## Discussion

DSRCT is a highly malignant tumor that occurs in young men aged 20–30 years [1]. The average age at onset is 22 years in male individuals, which tends to be younger in female individuals (20 years) [16]. In our study, all patients were male with a median age of 30 years. All tumors of our study occurred in common locations. Most patients presented with typical symptoms of abdominal distension, abdominal pain. All symptoms were consistent with those reported in published literature [14, 17].

Regarding gross pathology, tumors are generally large in size with uneven morphology, and gray surfaces that are visible after dissection, and are accompanied by peritoneal seeding, focal necrosis, mucus, or cystic formation. In terms of histopathology, multidirectional differentiation is prominent. The proportions of tumor cells and fibrous tissue vary in different regions. Tumor cells that we have observed were small to medium in size and round or oval in shape and had sparse cytoplasm, ambiguous borders, deep-blue stained nuclei, and inconspicuous nucleoli. Pyknosis and apoptosis were also visible separated by fibrous tissue into nests of different sizes with clear boundaries. In terms of IHC features, tumor cells were generally positive for WT-1 C-terminal, while only 2 patients were focally positive for WT1 N-terminal. The translocation between *WT1* and *EWSR* genes produces a gene fusion composed of the N-terminal of *EWSR* (the first 7 exons) and the C-terminal of *WT1* (the last 3 exons), which in turn results in a chimeric transcript [18]. The presence of the C-terminal-encoding domain of WT1 in the fusion transcript may be demonstrated by IHC, whereas the N-terminal region of WT1 is usually negative in DSRCT because it is lost in the fusion protein [19]. Therefore, IHC of WT1 should be interpreted carefully with the knowledge on the antibody specificity and IHC on C-terminal of WT1 was suggested to increase the diagnosis accuracy of DSRCT. Vimentin and desmin are also expressed in paranuclear dot-like patterns [20, 21]. In our series, 6 patients were positive for CK and 5 were positive for CD99, whereas 7 patients were positive for EMA, desmin, and vimentin.

In terms of molecular pathology, detecting the *EWSR1-WT1* gene fusion using FISH was critical for definitive diagnosis. More than 90% of the reported cases had typical t(11; 22)(p13; q12) translocation, resulting in the fusion between *EWSR1* and *WT1* genes on chromosome 11p13 [22]. In our study, we used a dual fusion FISH probe but not the break-apart probes for *EWSR1* to detect *EWSR1-WT1* fusion and found that all cases were *EWSR1-WT1* gene fusion positive. It should be noted that the break-apart probes for *EWSR1*, although being more commonly used, lack the specificity for DSRCT diagnosis. These probes show rearrangements in most other neoplasms with *EWSR1* gene fusion and could not determine the genes that are fused with *EWSR1* [23].

DSRCT should be distinguished from other small round cell malignancies, including extraskeletal Ewing sarcoma or primitive neuroectodermal tumor (PNET), small-cell carcinoma, neuroblastoma, rhabdomyosarcoma, malignant mesothelioma, and gastrointestinal stromal tumor (GIST). Ewing sarcomas/PNETs show CD99-positive staining in the cytoplasm and not in the cell membrane and are negative for CK, EMA, WT1, and desmin. Most importantly, they are negative for *EWSR1-WT1*. Small-cell carcinomas are positive for IHC, CK, TTF1, and neuroendocrine markers but negative for desmin. In addition to SMA and desmin, alveolar and embryonic rhabdomyosarcoma express MyoD1 and myogenin but not CK or neuroendocrine markers. Neuroblastomas are positive for neuroendocrine markers and negative for CK, WT1, and desmin. Malignant mesotheliomas have no characteristic paranuclear dot-like expressions of desmin and vimentin. Nevertheless, they can be positive for WT1. GISTs mostly express CD117, Dog-1, and CD34. Among our patients, 6 were CK positive and 7 were EMA positive, allowing the exclusion of Ewing sarcoma/PNET and small-cell carcinoma. Our cases were negative for CD117, CD34, and Dog-1, which excluded GIST. Acinar and embryonic rhabdomyosarcoma were excluded without rhabdomyoid cells. Malignant mesothelioma and neuroblastoma were also excluded because desmin-positive nuclei were present.

There is no consensus regarding the optimal therapy for DSRCT. CRS has been considered the mainstay and is defined as definite removal of at least 90% of tumor burden. Despite CRS combined with chemotherapy, abdominal radiotherapy, HIPEC, and even targeted therapy, overall antitumor effects have been unsatisfactory. Survival rates vary among different schemes. Preceding CRS with chemotherapy, possibly by effectively reducing tumor volume and anti-angiogenesis, has enabled a 3-year survival rate in 58% of patients [5]. Another study that involved 26 DSRCT patients who underwent complete and incomplete resection after neoadjuvant chemotherapy reported a median survival time of 63 and 26 months, respectively, suggesting that the extent of tumor reduction directly influenced survival time [24]. Honoréa et al. followed up 100 patients with DSRCT from 23 to 311 months (median: 25 months) [25, 26] and reported that CC0-1 or median PCI <12 indicated good prognosis, while HIPEC failed to improve survival. Postoperative whole abdominal radiotherapy was an independent prognostic factor. Gani et al. reported a mortality rate of 69.7% in 491 patients, and a median survival time of 25.9 months [27]. Multivariate analysis revealed that clinical stage, surgical treatment (regardless of surgical method), and adjuvant radiotherapy or chemotherapy were independent prognosis factors. Therefore, preoperative chemotherapy, CRS, and postoperative chemotherapy and radiation play vital roles in improving overall survival. The importance of HIPEC, however, needs to be further explored. In our study, one patient underwent right hemicolectomy and omental resection. Five patients were administered preoperative chemotherapy and 6 were treated with CRS plus HIPEC, of whom 3 patients had CC0-1, suggesting satisfactory depletion of tumor cells. However, PCI scores in 7 patients were >12, indicating a poor prognosis. Five patients were treated with postoperative chemotherapy and followed up for 7.5–28.5 months. Three patients died at 8.9, 14.4, and 28.5 months post-operation. The short survival time might be because of poor general conditions on admission, severe ascites, and lack of preoperative chemotherapy. Owing to the limited number of cases involved, the significance of HIPEC could not be assessed. The remaining 5 patients have been closely followed up.

Owning to limited researches, accurate diagnosis of DSRCT is challenging in clinic. Besides, there is no consensus on the treatment strategy. Our study provided a detailed review of the clinicopathological features and prognosis of 8 patients, which might provide help for the diagnosis and treatment of DSRCTs. A limitation should be noted in this study. An NGS-based PCR approach could be a helpful method for accurately diagnosing DSRCT. The sensitivity of this method is higher than that of FISH and RT-PCR. This method was not applied in this study because the RNA in our formalin-fixed and paraffin-embedded tissues might have been degraded. However, FISH assay for *EWSR1*, interpreted together with morphological and IHC findings, could also generate a relative definite diagnosis in this study.

## Conclusion

We described clinicopathological features, diagnosis, and treatment of 8 patients with DSRCT in the abdominal cavity. Most of the tumors were at the advanced clinical stage, with characteristic pathological morphology, IHC phenotype, and molecular biology profile. We recommend a comprehensive treatment approach consisting of preoperative chemotherapy, CRS with simultaneous HIPEC, and postoperative chemoradiotherapy. PCI and CC scores should be evaluated to assess therapeutic effects. Testing for combined expression of desmin, cytokeratins, and C-terminal of WT-1, as well as morphologic features, might be helpful during DSRCT diagnosis. The *EWSR1-WT1* gene fusion is the gold standard for definitive diagnosis. Further investigations involving a larger sample size are warranted to explore new treatment methods and improve survival rates in patients with DRSCT.

## Data Availability

All data generated or analyzed during this study are included in this published article.

## Abbreviations

DSRCTs: Desmoplastic small round cell tumors
CRS: Cytoreductive surgery
HIPEC: Hyperthermic intraperitoneal chemotherapy
PCI: Peritoneal cancer index
CC: Completeness of cytoreduction
CK: Cytokeratin
EMA: Epithelial membrane antigen
Syn: Synapsin
FISH: Fluorescence in situ hybridization
PNET: Primitive neuroectodermal tumors
GIST: Gastrointestinal stromal tumor
IHC: Immunohistochemical
